# Senescence-associated secretory phenotype: the “pathogenic” factor driving orthopedic degenerative diseases and its regulation

**DOI:** 10.3389/fragi.2026.1818021

**Published:** 2026-05-18

**Authors:** Junxiao Ren, Xiangjin Wang, Xin Zhou, Xiaohong Fan

**Affiliations:** Hospital of Chengdu University of Traditional Chinese Medicine,Chengdu, Sichuan, China

**Keywords:** cellular senescence, cellular senescence (CS), inflammation, orthopedic degenerative diseases, senescence-associated secretory phenotype

## Abstract

Orthopedic degenerative diseases, including osteoarthritis (OA), intervertebral disc degeneration (IVDD), and osteoporosis (OP), are major causes of chronic pain and functional decline in aging populations worldwide. The senescence-associated secretory phenotype (SASP), a downstream but central effector of cellular senescence, has emerged as a key pathogenic mediator that links senescent cell accumulation to tissue degeneration in the musculoskeletal system. Comprising pro-inflammatory cytokines, chemokines, matrix-degrading enzymes, growth factors, and extracellular vesicle-associated signals, SASP disrupts orthopedic tissue homeostasis through several interconnected mechanisms, including chronic sterile inflammation, extracellular matrix (ECM) catabolism, paracrine senescence propagation, stem/progenitor cell dysfunction, and, in selected contexts, aberrant neurovascular remodeling. In this review, we focus on the tissue-specific roles of SASP in major orthopedic degenerative diseases and organize current evidence according to mechanism-to-disease and mechanism-to-therapy relationships. We further summarize mechanism-linked therapeutic strategies, including senolytics, senomorphics, autophagy-based interventions, and emerging gene-/RNA-targeted approaches, while distinguishing between established preclinical avenues and exploratory modalities. Finally, we highlight key barriers to clinical translation, including tissue heterogeneity, biomarker selection, delivery specificity, safety, and trial design, to provide a more clinically actionable framework for future mechanistic and translational research.

## 1 Introduction

Orthopedic degenerative disorders, such as osteoarthritis (OA), intervertebral disc degeneration (IVDD), and osteoporosis (OP), constitute a significant and growing global health challenge in aging populations (Falvino et al., 2024; Bellini et al., 2020; Liu Y. et al., 2024). Previously regarded mainly as passive “wear-and-tear” conditions, these diseases are now recognized as dynamic, cell-driven processes influenced by the gradual aging of skeletal tissues. A key element of this conceptual shift is cellular senescence—a stable state of cell cycle arrest—and its most functionally impactful downstream characteristic, the senescence-associated secretory phenotype (SASP) (Noh et al., 2024; Zhu et al., 2022). It is critical to note that the SASP is not the trigger of cellular senescence itself; instead, it serves as a downstream yet pathogenic effector program through which senescent cells alter the local microenvironment and contribute to age-related tissue impairment (Coppé et al., 2010).

The SASP consists of a complex, biologically active secretome secreted by senescent cells, including proinflammatory cytokines, chemokines, matrix-degrading enzymes, and growth factors (Noh et al., 2024; Zhao et al., 2020). In addition to aging, factors that initiate SASP activation in orthopedic tissues include oxidative stress, mitochondrial dysfunction, mechanical overloading, and stimulation by proinflammatory cytokines (e.g., IL-1β, TNF-α) (Yagi et al., 2023; Yin and Xu, 2022; Kong et al., 2023; Wang et al., 2025). While cellular senescence initially acts as a tumor-suppressive mechanism, the sustained accumulation of senescent cells (due to impaired immune clearance) and chronic SASP secretion convert this protective response into a key mediator of tissue dysfunction. Immune cells (e.g., macrophages, T cells, dendritic cells, and innate lymphoid cells) play critical roles in SASP amplification: SASP recruits immune cells to clear senescent cells, but age-related immunosenescence reduces clearance efficiency, forming a “SASP-immune dysfunction-senescence” vicious cycle (Rentscher et al., 2022; Faust et al., 2020; Yin et al., 2025). Macrophages, the key innate immune effector cells in orthopedic tissues, display phenotypic plasticity upon SASP stimulation: SASP activates pro-inflammatory M1 macrophages to secrete pro-inflammatory cytokines and matrix-degrading enzymes, exacerbating inflammation and ECM catabolism, while suppressing reparative M2 macrophages (Wang et al., 2025; Wu C. J. et al., 2022). CD4^+^ Th1 cells secrete IFN-γ to boost SASP in senescent chondrocytes and NP cells; CD8^+^ T cells lose cytotoxicity via NKG2D downregulation, failing to clear SASP-high senescent cells (Rousseau et al., 2025; King et al., 2025; Novais et al., 2025). DCs show age-related dysfunction, impairing SASP-antigen presentation and T cell activation. SASP chemokines recruit ILC3s to lesions, whose IL-17 and GM-CSF amplify the SASP inflammatory cascade, forming a positive feedback loop between immune infiltration and SASP secretion in bone, cartilage and intervertebral disc tissues (Elsayed et al., 2023; Li et al., 2025a). Within the musculoskeletal system, SASP functions as a “pathogenic” factor, enabling senescent cells to disrupt local tissue homeostasis through detrimental paracrine and even endocrine effects (Lagoumtzi and Chondrogianni, 2021; Song Y. et al., 2024). This chronic, low-grade inflammation fueled by SASP—referred to as “inflammaging”—promotes a catabolic state, impairs regenerative capacity, and ultimately leads to the progressive structural and functional deterioration characteristic of orthopedic degenerative diseases (Wu et al., 2019; Giunta et al., 2022; Cheng X. et al., 2025).

**FIGURE 1:**
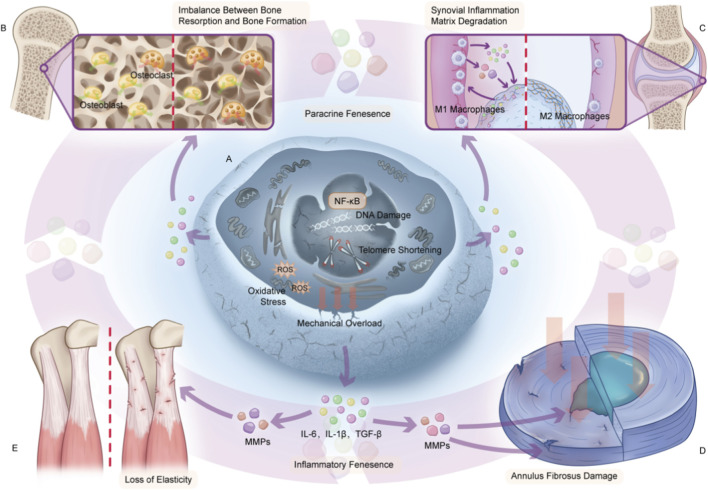
SASP links cellular senescence to orthopedic multisystem degeneration. **(A)** Endogenous/exogenous stresses (telomere shortening, DNA damage, etc.) activate NF-κB, inducing senescence; senescent cells secrete SASP (IL-6, IL-1β, MMPs, ADAMTS), forming a pathogenic microenvironment. **(B)** Bone: SASP disrupts resorption-formation balance (activates osteoclasts, inhibits osteoblasts), causing OP. **(C)** Joints: SASP induces synovial macrophage M1 polarization, releasing factors/MMPs to degrade cartilage collagen/proteoglycans, forming a “synovitis-cartilage degeneration” cycle, promoting OA. **(D)** Discs: SASP upregulates MMPs, damaging NP (reduces proteoglycan synthesis, dehydrates) and AF (collagen rupture), leading to herniation. **(E)** Ligaments/tendons: SASP degrades collagen via MMPs, causing disorders/tears, reducing strength, inducing tendinopathy/ligament laxity. Notes: Created by Junxiao Ren. This figure is original. Abbreviations: SASP (Senescence-Associated Secretory Phenotype), NF-κB (Nuclear Factor Kappa-Light-Chain-Enhancer of Activated B Cells), IL (Interleukin), TGF-β (Transforming Growth Factor-Beta), MMPs (Matrix Metalloproteinases), ADAMTS (A Disintegrin and Metalloproteinase with Thrombospondin Motifs), NP (Nucleus Pulposus), AF (Annulus Fibrosus).

The biological function of SASP exhibits a distinct “duality”: Under physiological conditions, short-term and acute SASP can contribute to embryonic development, wound healing, and tissue repai (Noh et al., 2024; Li et al., 2023); however, under pathological conditions, chronic and persistent SASP forms a “senescence spreading” chain through “autocrine-paracrine” effects—it not only induces adjacent healthy cells to enter a senescent state, but also remodels the local microenvironment and exacerbates inflammatory responses as well as tissue dysfunction (Li et al., 2023; Manni et al., 2026). This pathological effect is particularly prominent in orthopedic tissues: For instance, SASP secreted by senescent osteocytes can impair the mechanosensory capacity of bone tissue (e.g., IL-6 downregulates the P2X Purinoceptor 7(P2X7) receptor to weaken the mechanosensory response of osteocytes) (Gardinier et al., 2022); SASP from senescent bone marrow mesenchymal stem cells (BMSCs) (e.g., C-C Motif Chemokine Ligand 5(CCL5), IL-6) promotes osteoclast differentiation while inhibiting their own osteogenic differentiation capacity, leading to bone loss (Wang Y. et al., 2023; Yang et al., 2024); SASP released by senescent chondrocytes directly degrades type II collagen and proteoglycans (e.g., MMP-13, ADAMTS-4/5), and stimulates synovial macrophages to polarize toward the proinflammatory M1 phenotype, forming a “cartilage degeneration-synovial inflammation” vicious cycle (Yagi et al., 2023; Zhou et al., 2025); Similarly, SASP from intervertebral disc cells (nucleus pulposus (NP), annulus fibrosus(AF), and endplate cells) disrupts matrix homeostasis (by inhibiting the synthesis of type II collagen and aggrecan, and promoting the secretion of matrix-degrading enzymes), thereby serving as a key driver of IVDD(3, 32, 33). More importantly, the interaction between SASP and the immune system further amplifies the pathological progression of orthopedic degenerative lesions. On one hand, SASP can recruit immune cells such as macrophages and T cells to clear senescent cells, maintaining tissue homeostasis (Ling et al., 2025; Han et al., 2024); On the other hand, age-related “immunosenescence” (e.g., impaired proliferative capacity of immune cells, dysregulated cytokine secretion) reduces the efficiency of senescent cell clearance. This leads to the sustained accumulation of SASP, which in turn accelerates the senescence of immune cells, forming a vicious cycle of “inflammation-senescence-immune dysfunction”—namely “inflammaging” (Noh et al., 2024; Li et al., 2023). In orthopedic diseases, this cycle manifests as follows: SASP induces M1 polarization of macrophages to exacerbate cartilage destruction, regulates the phenotype of bone marrow immune cells to disrupt bone metabolism,and even intensifies pain perception by promoting vascular and nerve ingrowth into intervertebral disc tissue, ultimately acting as a “booster” for disease progression (Figure 1) (Zhou et al., 2025; Peng et al., 2025; Mannarino et al., 2025). Despite rapid advances in SASP research in musculoskeletal aging, several important questions remain unresolved, including how SASP composition differs across orthopedic tissues, which downstream mechanisms dominate in specific diseases, and which intervention strategies most directly interrupt those mechanisms. Therefore, this review is organized in a stepwise manner: we first define the orthopedic relevance of cellular senescence and SASP, then summarize their tissue-specific manifestations in bone, cartilage, intervertebral disc, and periarticular soft tissues, next dissect the principal pathogenic mechanisms through which SASP promotes degeneration, and finally align disease-specific therapeutic strategies with those mechanisms and discuss their translational potential (Lagoumtzi and Chondrogianni, 2021; Lim et al., 2022; Song H. et al., 2024; Cherif et al., 2020).

## 2 The manifestation of cellular senescence and SASP in orthopedic tissues

### 2.1 The relationship between cellular senescence and SASP

Cellular senescence and SASP are linked in a hierarchical rather than equivalent manner. Cellular senescence refers to a durable stress-response state characterized by cell-cycle arrest and altered metabolic and transcriptional activity, whereas SASP is the major downstream secretory program through which senescent cells influence neighboring cells and tissue homeostasis (López-Otín et al., 2023; Olinger et al., 2025; Alqahtani et al., 2025). In the orthopedic context, this distinction is especially important because disease progression is driven less by growth arrest alone than by the persistence of a pathogenic secretome composed of inflammatory mediators, matrix-remodeling enzymes, growth factors, and extracellular vesicle-associated signals. Accordingly, the clinical relevance of senescent cells in bone, cartilage, and intervertebral disc tissues lies chiefly in their capacity to establish a chronic catabolic microenvironment through SASP output rather than in senescence as an isolated intracellular state.

Under physiological conditions, transient senescence can contribute to tissue remodeling and repair, and a short-lived SASP may help coordinate immune surveillance and damage resolution (Chang et al., 2016; Chung et al., 2019; Abo Qoura et al., 2026; Chen et al., 2025). In contrast, under pathological conditions relevant to orthopedic aging—such as chronic mechanical overload, oxidative stress, persistent inflammatory stimulation, and impaired immune clearance—senescent cells accumulate and maintain prolonged SASP secretion through DDR-, NF-κB-, p38-MAPK-, and related signaling pathways (Yin and Xu, 2022; Xia et al., 2025; Franceschi et al., 2018; Salminen et al., 2012; Xu et al., 2024). This chronic SASP state reinforces senescence in an autocrine manner and spreads senescence to adjacent cells through paracrine signaling, thereby amplifying inflammation, matrix catabolism, and loss of tissue homeostasis (Xu et al., 2017; Cinat et al., 2021; Tang et al., 2025; Wu D. et al., 2025). In orthopedic tissues, this process is manifested by impaired osteocyte mechanosensation, chondrocyte-driven cartilage catabolism, disc cell-mediated ECM breakdown, and progressive dysfunction of local stem/progenitor cell pools, collectively providing the mechanistic basis for age-related musculoskeletal degeneration (Zhang et al., 2025; Li et al., 2025b; Farr et al., 2020).

### 2.2 Cellular senescence and SASP in skeletal tissues (bone, cartilage and intervertebral disc)

Cellular senescence and the subsequent SASP are conserved pathological drivers of degeneration in bone, cartilage, and intervertebral disc (IVD) tissues—core skeletal components affected by age-related orthopedic diseases—with shared senescent cell characteristics and SASP-mediated pathogenic mechanisms, alongside tissue-specific triggers and functional consequences (Liu Y. et al., 2024; Yagi et al., 2023; Gardinier et al., 2022; Novais et al., 2019). Across these three tissues, senescent cells universally exhibit upregulated p16/p21 expression, accumulated DNA damage (γH2AX positivity), telomere shortening, enhanced SA-β-galactosidase activity, and cell cycle arrest, with senescence triggered by overlapping stimuli including mechanical overload, inflammatory stress (e.g., IL-1β, TNF-α), oxidative stress, and radiation, as well as tissue-specific inducers such as cadmium and estrogen/vitamin D insufficiency (Yin and Xu, 2022; Kong et al., 2023; Wang Y. et al., 2023; Yang et al., 2024; Wang et al., 2021; Wang J. et al., 2023).

In bone tissue, osteocytes, BMSCs, and osteoclasts are the primary senescent cell populations (Gardinier et al., 2022; Wang Y. et al., 2023; Wang et al., 2021). As the most abundant and long-lived bone cells, senescent osteocytes display impaired mechanotransduction capacity due to SASP (IL-6)-mediated downregulation of the P2X7 receptor, contributing to age-related bone loss (Buck and Stains, 2024; Al-Bari and Al, 2020). Senescent BMSCs exhibit diminished osteogenic differentiation and an enhanced adipogenic tendency, while their secreted SASP factors (CCL5, IL-6, IL-1β) promote osteoclast formation and bone resorption, exacerbating OP via disrupted bone formation-resorption balance (Wang Y. et al., 2023; Huo et al., 2024).

In cartilage tissue, articular chondrocyte senescence is the central driver of OA initiation and progression, with p16-positive senescent cell numbers and SASP release increasing with age and OA severity. Yagi et al. demonstrated that oxidative stress (H_2_O_2_) primarily induces chondrocyte p21 upregulation, Reactive Oxygen Species(ROS) accumulation, and reduced glycosaminoglycan (GAG) synthesis, while inflammatory stimuli (IL-1β/TNF-α) drive p16 expression and robust SASP secretion, both ultimately causing cartilage matrix degradation (Yagi et al., 2023). Senescent chondrocyte-derived SASP (IL-1β, IL-6, TNF-α) and matrix-degrading enzymes (MMPs, ADAMTS) directly degrade type II collagen and proteoglycans, and also induce synovial macrophage M1 polarization to form an “inflammation-senescence” vicious cycle, amplifying cartilage degeneration (Peng et al., 2025; Chen et al., 2023; Zhao et al., 2023).

In the intervertebral disc (IVD), comprising NP, AF, and cartilaginous endplate (EP) regions, NP and AF cells are the main senescent cell targets, with aging leading to increased p16/p21-positive cells and reduced Ki67-positive proliferating cells (Novais et al., 2019; Du et al., 2022). Senescent NP cells secrete SASP factors (IL-1β, IL-6, IL-8, MMP-3/13, ADAMTS) that suppress type II collagen/aggrecan synthesis and promote matrix catabolism, disrupting IVD homeostasis (Liu Y. et al., 2024; Lim et al., 2022; Wu Y. et al., 2022; Peng et al., 2020). Tissue-specific senescence inducers include cadmium, which triggers AF cell senescence via the JNK/p53 pathway (upregulating p16/p21 and IL-1β/IL-6 release), and oxidized LDL, which induces EP chondrocyte senescence and calcification through the LOX-1/ROS/P38-MAPK/NF-κB axis (Liu X. et al., 2024; Bing et al., 2024). Senescent NP cells further drive IVDD via SASP-mediated paracrine crosstalk with endothelial cells: SASP factors (VEGF, IL-6, bFGF) promote vascular ingrowth into the avascular IVD microenvironment, and activated endothelial cells in turn secrete Ang-1/PDGF-BB to amplify NP cell SASP expression and ECM degradation, forming a degenerative feedback loop (Song H. et al., 2024; Wu Z. et al., 2025; Zhu et al., 2025). Collectively, SASP secreted by senescent cells in bone, cartilage, and IVD tissues exerts conserved pathogenic effects including ECM catabolism, inflammatory amplification, and paracrine senescence spreading, with tissue-specific molecular and cellular interactions shaping the distinct clinical manifestations of OP, OA, and IVDD.

### 2.3 Cellular senescence and SASP in ligament and tendon tissues

Evidence for cellular senescence in ligament and tendon tissues is less extensive than that for bone, cartilage, and intervertebral disc, but available data support a contributory role in age-related loss of joint stability and impaired soft-tissue healing (Stowe et al., 2024; Korcari et al., 2023; Nakamichi and Asahara, 2021). In these tissues, senescence is associated with reduced fibroblast or tenocyte proliferative capacity, diminished collagen synthesis, disorganized matrix remodeling, and increased expression of inflammatory and matrix-degrading mediators, changes that may weaken tensile properties and delay enthesis repair (Stowe et al., 2024; Korcari et al., 2023). Tendon and enthesis aging are particularly relevant to osteoarthritis because impaired tendon-bone integration and periarticular soft-tissue insufficiency can alter joint biomechanics and secondarily accelerate cartilage degeneration (Greif et al., 2020). Moreover, emerging evidence indicates that senescent tenocytes and ligament fibroblasts exhibit a pronounced pro-inflammatory and catabolic SASP profile, characterized by elevated secretion of IL-1β, IL-6, TNF-α, and matrix-degrading enzymes (Hsu et al., 2024; Chen et al., 2022). This SASP further disrupts collagen fibrillogenesis, weakens tissue tensile strength, and perpetuates a chronic inflammatory microenvironment that impairs endogenous repair. Notably, SASP derived from aged tendon and ligament cells can propagate senescence to adjacent healthy cells via paracrine signaling, establishing a self-sustaining degenerative loop that compromises enthesis integrity and joint stability. Such paracrine effects not only exacerbate local soft-tissue deterioration but also contribute to the pathogenesis of adjacent joint structures, linking tendinopathy and ligamentous insufficiency to the initiation and progression of osteoarthritis (Varela-Eirín et al., 2022; Wang et al., 2022) (Table 1).

**TABLE 1:** Tissue-specific characteristics of cellular senescence and SASP in orthopedic tissues.

Orthopedic tissue	Primary senescent cell populations	Key SASP factors	Tissue-specific senescence inducers	Pathological consequences of SASP	Corresponding references
Bone	Osteocytes, bone marrow mesenchymal stem cells (BMSCs), and osteoclast-lineage cells	IL-6, CCL5, IL-1beta, and other osteoclast-supportive mediators	Estrogen deficiency, active vitamin D insufficiency, oxidative stress, irradiation, and age-related mechanical dysregulation	Impaired osteocyte mechanosensation, reduced osteogenic differentiation of BMSCs, enhanced osteoclastogenesis and bone resorption, and disturbed bone remodeling leading to osteoporosis	28, 29, 30, 58,59,60,61,62,63
Cartilage	Articular chondrocytes	IL-1beta, IL-6, TNF-alpha, MMPs, and ADAMTS	Oxidative stress, inflammatory cytokine stimulation, and mechanical overloading	Type II collagen/proteoglycan loss, amplification of synovial inflammation through macrophage M1 polarization, and self-reinforcing cartilage degeneration in osteoarthritis	8, 36, 64, 65
Intervertebral disc (NP/AF/EP)	Nucleus pulposus cells, annulus fibrosus cells, and endplate chondrocytes	IL-1beta, IL-6, IL-8, MMP-3/13, ADAMTS, VEGF, and bFGF	Cadmium exposure, oxidized LDL, inflammatory stress, and mechanical overload	Suppressed type II collagen/aggrecan synthesis, accelerated ECM catabolism, endothelial/vascular ingrowth into the normally avascular disc, and progressive structural degeneration in IVDD	3, 32, 33, 38, 39, 66, 67, 68, 69, 70, 71
Ligament/Tendon	Tenocytes and ligament fibroblasts	IL-1beta, IL-6, TNF-alpha, and matrix-degrading enzymes	Aging-related mechanical unloading/instability and chronic degenerative stress	Reduced cell proliferation and collagen synthesis, disorganized matrix remodeling, impaired enthesis repair, loss of tensile strength, and propagation of periarticular degeneration contributing to joint instability and osteoarthritis progression	72, 73, 74,75, 76, 77,78,79

## 3 Mechanisms of SASP-Driven orthopedic degenerative lesions

The SASP promotes the progression of orthopedic degenerative diseases via a set of interconnected and self-amplifying pathological mechanisms. Together, these mechanisms create a degenerative microenvironment that impairs tissue structure and function (Catheline et al., 2021; Gries et al., 2022). Promotion of Chronic Inflammation and Immune DysregulationThe primary pathogenic mechanism of SASP is the instigation and perpetuation of a persistent, low-grade inflammatory state (Gries et al., 2022; Sebastian-Valverde and Pasinetti, 2020). With the accumulation of senescent cells, a large number of pro-inflammatory mediators—including IL-1β, IL-6, TNF-α, and C-C motif chemokine ligand 2 (CCL2)—are secreted. These mediators engage autocrine and paracrine signaling loops to activate the NF-κB pathway, thereby forming a feedforward cycle that further amplifies inflammatory responses (Faust et al., 2020; Wu et al., 2019). This inflammatory cascade does not merely cause local cytokine elevation but also affects systemic inflammatory networks. Notably, SASP components can initiate complement system activation, which eventually leads to the assembly of membrane attack complexes (MAC). While this mechanism has been well documented in choroidal capillary degeneration, growing evidence suggests that it also participates in orthopedic tissue injury (Blasiak, 2020; Vun et al., 2023). A key aspect of SASP’s pro-inflammatory activity is its regulation of immune cell functions. The SASP recruits and functionally modifies multiple immune cell populations: for example, in post-traumatic osteoarthritis, IL-17, a major SASP factor, enhances joint inflammation by attracting innate lymphoid cells, γδ+ T cells, and CD4^+^ T cells to the affected tissues (Faust et al., 2020). Moreover, the SASP exerts a strong effect on macrophage polarization, converting these cells from the anti-inflammatory, tissue-reparative M2 phenotype to the pro-inflammatory, matrix-degrading M1 phenotype (Zhou et al., 2025; Peng et al., 2025; Kang et al., 2021). Therapeutic strategies aimed at reducing SASP activity—such as adjusting the STAG1/TP53/P21 pathway and using Imrecoxib for pharmacological inhibition of the cyclooxygenase-2/prostaglandin E2 (COX-2/PGE2) axis—can reverse this polarization, reduce inflammation, and improve disease progression. These discoveries emphasize the central role of SASP-driven inflammation in the degenerative process (Zhou et al., 2025; Peng et al., 2025).

In addition to its pro-inflammatory effects, the SASP directly impairs the structural integrity of tissues by disrupting the homeostasis of the extracellular matrix (ECM) (Yan et al., 2024; Vamvakas et al., 2017). Senescent cells generate a range of matrix-degrading enzymes, such as MMP-3, MMP-13, and ADAMTS-4/5, which exert proteolytic cleavage on key structural ECM components like collagen and proteoglycans (Zhao et al., 2023). To exacerbate this catabolic damage, the SASP also downregulates the expression of genes essential for *de novo* ECM synthesis, including collagen type II alpha 1 chain (COL2A1) and aggrecan (ACAN). This dual effect impairs the inherent reparative capacity of tissues (Feng et al., 2021). This overall imbalance between ECM degradation and synthesis has been observed in various orthopedic tissues. In articular cartilage, natural products like dendrobine have been proven to alleviate OA progression by blocking the ROS/NF-κB signaling axis, which in turn reduces the production of SASP factors and the subsequent release of MMP-13 and ADAMTS-5(64). In a similar fashion, traditional herbal formulations that target pathways including STAG1/TP53/P21 can suppress SASP activity and exert protective effects on articular cartilage (Zhou et al., 2025). In the intervertebral disc, maintaining ECM homeostasis requires complex regulatory networks; for instance, promoting the clearance of Serine-Rich RNA Binding Domain 1(SRBD1) via the autophagic receptor Next to BRCA1 Gene 1(NBR1) or activating chaperone-mediated autophagy (CMA) through UCHL1-mediated deubiquitination of Heat Shock Protein Family A (Hsp70) Member 8(HSPA8) can counteract NP cell senescence and SASP production, thereby alleviating ECM degradation (Song H. et al., 2024; Wu Z. et al., 2025).

The catabolic activity of SASP sets in motion a deleterious feed-forward cycle (Wang and Guan, 2025). The ECM degradation products (ECMDPs) generated by SASP-derived enzymes—such as collagen fragments, proteoglycan breakdown products, and matrikines—are themselves biologically active. These ECMDPs bind to pattern recognition receptors (e.g., TLR2/4) on senescent and neighboring cells, activating downstream pathways like MyD88/NF-κB to further upregulate the transcription of pro-inflammatory SASP factors (IL-1β, IL-6) and matrix-degrading enzymes (MMP-13, ADAMTS-5). In disc tissue, fragmented fibronectin and laminin can activate the integrin β1/FAK/p38-MAPK pathway, enhancing SASP production and accelerating NP cell senescence (Baylie et al., 2026; Giroud et al., 2023). Similarly, in bone, osteocalcin fragments and collagen I peptides can amplify SASP in osteocytes and BMSCs via pathways like Ca^2+^/NFATc1, further destabilizing the bone remodeling balance (Ahmadi and Rezaie, 2021; Yang et al., 2025; He et al., 2023). This positive feedback loop, where SASP begets ECM damage which in turn begets more SASP, creates a self-sustaining engine for disease progression.

Beyond damaging the matrix, SASP acts as a vehicle for transmitting the senescent state to neighboring healthy cells, a process termed “senescence spreading” (Wu et al., 2019; Klepacki et al., 2025). SASP factors, often packaged within small extracellular vesicles (sEVs), act on adjacent cells to activate the p53/p21 and p16/RB pathways, inducing them to enter senescence and become new sources of SASP, thereby creating a chain reaction. For instance, OA chondrocytes release Cx43-enriched Small Extracellular Vesicles (sEVs) that induce senescence in target chondrocytes, synovial cells, and osteocytes via NF-κB and Extracellular Signal-Regulated Kinase 1/2(ERK1/2) signaling, fostering a degenerative microenvironment and promoting processes like chondrocyte dedifferentiation (Varela-Eirín et al., 2022). In the intervertebral disc, SASP from senescent NP cells exerts potent paracrine effects, and targeting this spread with senolytics like MDM2 Inhibitor RG-7112(RG-7112) and o-Vanillin can reduce senescent cell burden and improve matrix homeostasis (Cherif et al., 2020). Furthermore, pathways such as Cytokine Receptor-Like Factor 1/Cardiotrophin-Like Cytokine Factor 1(CRLF1/CLCF1) acting through Janus Kinase/Signal Transducer and Activator of Transcription(JAK/STAT3) can enhance SASP production and accelerate senescence spread in disc cells (Zhu et al., 2025). Halting this paracrine spread is therefore a critical therapeutic objective.

A major downstream effect of persistent SASP activity is the breakdown of tissue maintenance and regenerative capacity. The integrity of orthopedic tissues relies not only on mature structural cells but also on resident stem/progenitor cells, including bone marrow mesenchymal stem cells, skeletal stromal cells, and cartilage- or disc-supporting progenitor-like cells. The self-renewal, lineage commitment, and reparative functions of these cells are highly sensitive to the SASP-rich microenvironment (Wang Y. et al., 2023; Yang et al., 2024; Wang and Guan, 2025; Yang et al., 2025; He et al., 2023). In bone, the SASP contributes to mesenchymal stem cell exhaustion, reduced osteogenic differentiation, and enhanced osteoclast-supportive signaling; in cartilage, it suppresses the activity of reparative cells and limits matrix restoration; and in the intervertebral disc, it impairs endogenous repair while maintaining a catabolic microenvironment. Additionally, certain SASP factors—including VEGF, IL-6, and other pro-angiogenic or neurotrophic mediators—can promote abnormal vascular and nerve ingrowth, particularly in degenerating discs and osteochondral interfaces, thereby linking structural degeneration to the development of chronic pain (Mannarino et al., 2025; Song H. et al., 2024; Chen et al., 2026) (Table 2). Notably, emerging evidence indicates that gene editing and miRNA-mediated regulation serve as upstream molecular switches that directly modulate core SASP regulatory networks, including the p53/p21, NF-κB, and JAK/STAT pathways.

**TABLE 2:** Key pathogenic mechanisms of SASP in driving orthopedic degenerative diseases.

Pathogenic mechanism	Molecular and cellular events	Orthopedic tissue manifestations	Synergistic positive feedback loops	Corresponding references
Chronic inflammation and immune dysregulation	SASP-derived IL-1beta, IL-6, TNF-alpha, and CCL2 sustain NF-kappaB-centered inflammatory signaling, promote macrophage M1 polarization, recruit immune cells, and impair effective clearance of senescent cells	Synovitis and macrophage-driven cartilage injury in OA, inflammatory bone marrow microenvironment in OP, and a persistent inflammatory niche in IVDD	SASP accumulation →ineffective immune clearance/immunosenescence →further persistence of senescent cells and SASP (“inflammaging”)	13, 23, 31, 36
Extracellular matrix (ECM) homeostasis disruption	SASP-associated MMPs and ADAMTS catabolize collagen and proteoglycans while suppressing COL2A1 and ACAN expression, thereby shifting tissues toward a sustained catabolic state	Cartilage matrix erosion in OA, loss of disc ECM homeostasis in IVDD, and imbalance of bone matrix remodeling in OP	SASP → ECM degradation → ECM degradation products (ECMDPs) →TLR/NF-kappaB activation →more SASP and matrix-degrading enzymes	39, 64, 65, 70, 86, 87, 88
Senescence spreading	SASP factors, including extracellular vesicle-associated signals, induce p53/p21- and p16/RB-related senescence programs in neighboring cells through paracrine/autocrine signaling	Spread of senescence among chondrocytes and synovial cells in OA, propagation of NP-cell senescence in IVDD, and broader expansion of dysfunctional senescent cell networks in skeletal tissues	Senescent cells →pathogenic SASP release→adjacent-cell senescence →secondary SASP production →amplified tissue-wide degeneration	40, 71, 78, 95
Impaired tissue regenerative potential	Persistent SASP suppresses the self-renewal, lineage commitment, and reparative function of resident stem/progenitor populations, especially MSC-like cells	Reduced osteogenic capacity in OP, compromised subchondral/stromal repair in OA, and impaired endogenous repair in IVDD	SASP-rich niche→stem/progenitor-cell dysfunction →failed tissue maintenance/repair →accumulation of further degeneration and SASP	29, 30, 89, 93, 94
Aberrant vascular and nerve ingrowth	SASP-associated VEGF, IL-6, bFGF, and related mediators stimulate endothelial activation, vascular invasion, and in selected contexts neurovascular remodeling linked to pain	Vascular ingrowth into the normally avascular disc in IVDD and neurovascular remodeling at degenerative osteochondral/disc interfaces	SASP →vascular/immune infiltration and pain-related remodeling→further local inflammation and SASP reinforcement	37, 39, 70, 96

## 4 The role of SASP in specific orthopedic degenerative diseases

Having outlined the core pathogenic mechanisms of SASP, the following subsections focus on how these mechanisms are weighted and manifested in individual diseases. Rather than re-describing all mechanisms in parallel, we emphasize the dominant compartment-specific consequences of SASP in OA, IVDD, and OP, as well as the therapeutic implications most directly linked to those disease contexts.

### 4.1 The role of SASP in OA

SASP drives OA progression through three interconnected pathological processes: cartilage degeneration, synovial inflammation, and subchondral bone remodeling, with cross-talk between these compartments amplifying the degenerative microenvironment (Peng et al., 2025; Zhao et al., 2023; Zhao et al., 2025). SASP factors (IL-1β, IL-6, TNF-α) from senescent chondrocytes activate the NF-κB/MAPK pathway to upregulate MMP-13/ADAMTS-4/5, directly degrading cartilage matrix, while mechanical stress exacerbates this process via MicroRNA-325–3p(miR-325–3p) downregulation and p53/p21 activation (Zhao et al., 2023; Afzal et al., 2025). IKKβ-NF-κB signaling in chondrocytes further modulates SASP secretion (p65 promotes, p50 inhibits), a key regulatory node for OA-like phenotype development (Catheline et al., 2021). Abnormal ECM metabolism and SASP form a vicious cycle, and natural compounds such as procyanidin B2 (PCB2) and Dendrobine alleviate OA by inhibiting chondrocyte senescence/SASP via the Nrf2/NF-κB and ROS/NF-κB axes, respectively (Chen et al., 2023; Cai et al., 2022).

SASP is also a central mediator of OA-associated synovitis: senescent synovial cell-derived SASP regulates macrophage polarization, and Imrecoxib and Cerium Oxide Nanoparticles(CeNP) mitigate synovial inflammation by shifting macrophages toward the M2 phenotype via COX-2/PGE2 inhibition and ROS/NF-κB suppression, respectively (Peng et al., 2025; Ren et al., 2023). Cx43-enriched extracellular vesicles from OA chondrocytes induce synovial cell senescence and SASP production, promoting synovial fibrosis via epithelial-mesenchymal transition (EMT)-like processes and further amplifying joint degeneration (Varela-Eirín et al., 2022).

In subchondral bone, SASP from senescent chondrocytes/synoviocytes diffuses across the bone-cartilage interface to disrupt osteocyte, osteoblast, and osteoclast function. Parathyroid Hormone (Falvino et al., 2024; Bellini et al., 2020; Liu Y. et al., 2024; Noh et al., 2024; Zhu et al., 2022; Coppé et al., 2010; Zhao et al., 2020; Yagi et al., 2023; Yin and Xu, 2022; Kong et al., 2023; Wang et al., 2025; Rentscher et al., 2022; Faust et al., 2020; Yin et al., 2025; Wu CJ. et al., 2022; Rousseau et al., 2025; King et al., 2025; Novais et al., 2025; Elsayed et al., 2023; Li et al., 2025a; Lagoumtzi and Chondrogianni, 2021; Song Y. et al., 2024; Wu et al., 2019; Giunta et al., 2022; Cheng X. et al., 2025; Li et al., 2023; Manni et al., 2026; Gardinier et al., 2022; Wang Y. et al., 2023; Yang et al., 2024; Zhou et al., 2025; Novais et al., 2019; Liu X. et al., 2024; Ling et al., 2025)(PTH(1–34)) ameliorates temporomandibular joint OA by reducing subchondral bone senescent cell accumulation and SASP via Smad3/p16ink4a inhibition, while SASP factors (IL-6, RANKL) promote osteoclast formation and bone resorption, disrupting subchondral bone structure (Zhao et al., 2025; Cui et al., 2020). Obesity-related OA exacerbates this process via abnormal lipid metabolism, which modulates chondrocyte senescence/SASP through p53-FOXO3 and drives excessive osteoclast differentiation via ferroptosis (Zhao et al., 2025). SASP also impairs subchondral bone MSC osteogenic differentiation, and the dasatinib + quercetin (DQ) senolytic combination restores MSC function, reducing bone loss and improving subchondral bone regeneration in OA(29).

### 4.2 The role of SASP in IVDD

SASP is the primary pathogenic driver of IVDD, with senescence of NP, AF, and EP cells and subsequent SASP secretion disrupting IVD matrix homeostasis and driving structural deterioration (Liu Y. et al., 2024; Zhang et al., 2022; Song C. et al., 2024). Novais et al. confirmed that p16Ink4a deletion in mouse IVDs does not abrogate senescence but reduces SASP secretion and preserves matrix homeostasis, demonstrating that SASP—rather than p16+ senescent cells alone—drives IVDD(32). Senolytic therapies have shown robust preclinical efficacy for IVDD: RG-7112 and o-Vanillin selectively eliminate senescent disc cells and reduce SASP to improve matrix homeostasis, while local delivery of ABT263 via Poly(Lactic-Co-Glycolic Acid)(PLGA) nanoparticles clears senescent cells, reduces inflammatory factors/MMPs, and restores IVD structure (Lim et al., 2022; Cherif et al., 2020).

Multiple molecular pathways regulate IVD senescence and SASP secretion: Cannabinoid Receptor Type 2(CB2R) activation delays NP cell senescence and inhibits SASP via the Adenosine Monophosphate-Activated Protein Kinase/Glycogen Synthase Kinase 3β(AMPK/GSK3β) pathway, the NLRX1-SLC39A7 complex modulates mitochondrial dynamics/autophagy to reduce SASP, and the autophagic receptor NBR1 clears SRBD1 to retard NP cell senescence and SASP production (Song H. et al., 2024; Du et al., 2022). The IVD microenvironment further amplifies SASP: fibronectin enhances NP cell contractility to activate YAP/NF-κB, increasing SASP secretion, while UCHL1 deubiquitinates HSPA8 to activate CMA, antagonizing autophagy-dependent ferroptosis and alleviating NP cell senescence/SASP (Wu Z. et al., 2025; Naha and Driscoll, 2024). These mechanistic insights confirm SASP as a promising therapeutic target for IVDD, with tissue-specific targeted delivery strategies critical for translating these findings clinically.

### 4.3 The role of SASP in OP

OP is characterized by a disrupted bone formation-resorption balance, and SASP is a core mediator of this imbalance via multi-level regulation of bone cell function and bone marrow microenvironment (Wang and Guan, 2025; Weng et al., 2025). Estrogen deficiency, a major OP risk factor, activates the JAK2/STAT3 pathway in BMSCs to increase inflammatory factor and SASP secretion (CCL5, IL-6, IL-1β), impairing osteogenic differentiation and promoting osteoclast formation (Wu et al., 2020). The DQ senolytic combination reverses this process by clearing senescent bone marrow cells, restoring BMSC function, and promoting bone regeneration in postmenopausal OP(29). Insufficient active vitamin D accelerates osteocyte senescence and SASP secretion, and the senolytic ABT263 selectively eliminates senescent osteocytes to reduce oxidative stress/DNA damage, increase bone formation, and decrease resorption (Yang et al., 2024).

SASP also regulates osteoclast formation via NF-κB-dependent mechanisms: Ataxia-Telangiectasia Mutated(ATM) kinase activates NF-κB to upregulate SASP and promote OP, while ATM deletion reduces NF-κB activity and senescence to improve bone function (Zhao et al., 2020; Chou et al., 2022). In periodontitis-associated bone loss, Activating Transcription Factor 3(ATF3) modulates senescence/SASP via the STAT3/ERK and p65/AP-1 pathways, further promoting osteoclast differentiation and bone resorption (Bae and Lee, 2025). Epigenetic regulation is an additional layer of SASP control in OP: Huo et al. reviewed that epigenetic modifications govern OP-related cellular senescence, with Sirtuin 6(SIRT6) deletion increasing p21, γH2AX, and IL-6 expression to enhance SASP burden and accelerate bone/disc degeneration (Huo et al., 2024; Ramteke et al., 2024). Together, these studies identify SASP as a unifying target for OP therapy, with senolytics and senomorphics (e.g., JAK inhibitors) showing great potential for restoring bone homeostasis (Yang et al., 2024; Wu et al., 2020).

## 5 Regulatory mechanisms and intervention strategies of SASP

### 5.1 Molecular regulatory network of SASP

**FIGURE 2:**
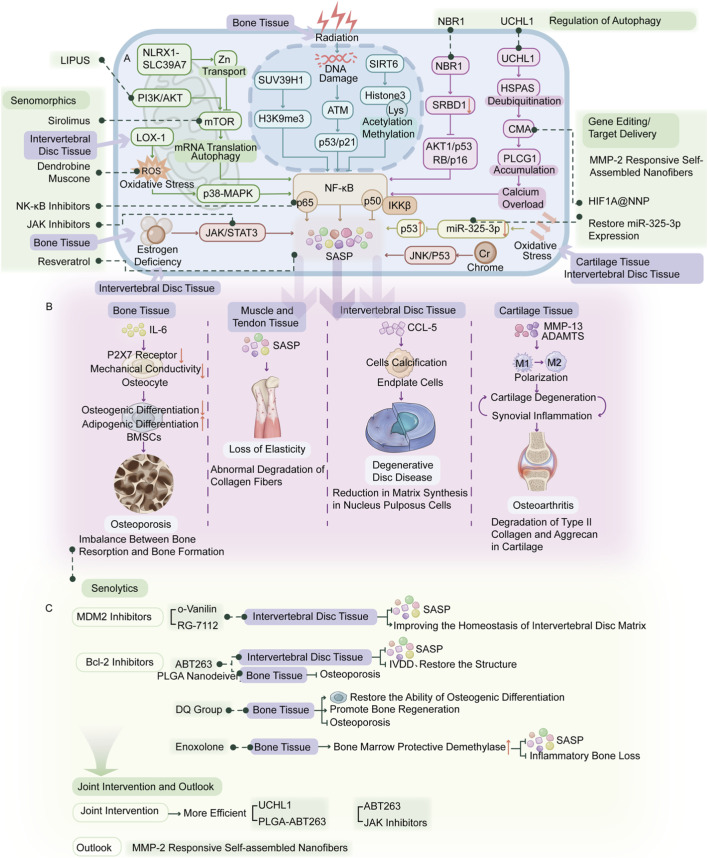
Molecular Regulatory Network and Therapeutic Intervention Strategies Targeting SASP in Orthopedic Degenerative Diseases. This figure summarizes the multilayered molecular regulation of SASP and corresponding mechanism-linked therapeutic approaches for orthopedic degeneration. **(A)** Core upstream signaling pathways governing SASP production and secretion in senescent orthopedic cells, including NF-κB, p38-MAPK, JAK/STAT, mTOR, and DNA damage response (DDR) pathways, which collectively determine SASP magnitude, composition, and persistence. **(B)** Tissue-specific SASP programs and pathogenic outputs in bone, cartilage, and intervertebral disc tissues, driving distinct degenerative phenotypes via ECM catabolism, immune dysregulation, senescence spreading, and stem cell dysfunction. **(C)** Classification and mechanistic targets of SASP-directed therapeutic strategies, including senolytics (senescent cell elimination), senomorphics (SASP suppression), autophagy modulation (proteostasis restoration), and gene/RNA-targeted precision interventions, aligned to interrupt specific SASP-driven pathological cascades. Notes:This regulatory-therapeutic framework highlights tissue heterogeneity in SASP regulation and supports the development of stratified, mechanism-based interventions to translate SASP-targeted strategies into clinical applications for OA, IVDD, OP, and other age-related musculoskeletal disorders.

Because SASP promotes orthopedic degeneration through definable downstream mechanisms, therapeutic strategies should be interpreted according to the mechanism(s) they primarily interrupt rather than as a simple list of anti-aging interventions. In broad terms, senolytics reduce the source of SASP by eliminating senescent cells; senomorphics suppress inflammatory and catabolic SASP output without necessarily killing the cells; autophagy-modulating approaches restore intracellular proteostasis and thereby attenuate senescence-associated secretory signaling; and gene-/RNA-targeted approaches aim to reprogram upstream regulators or disease-relevant effectors of SASP in a tissue-specific manner (Ling et al., 2025; Alqahtani et al., 2025; Chandra et al., 2020; Birch and Gil, 2020; Kang et al., 2026). This therapeutic logic reflects the fact that SASP is governed by a multilayered regulatory network rather than by a single linear pathway. In orthopedic tissues, DNA damage responses, oxidative stress, mitochondrial dysfunction, inflammatory cytokine exposure, and mechanical overload converge on core signaling nodes such as NF-κB, p38-MAPK, JAK/STAT, and mTOR, which together determine the magnitude, composition, and persistence of SASP output. These signals are further shaped by epigenetic regulators, non-coding RNAs, and autophagy-dependent quality-control systems, thereby contributing to tissue-specific differences in senescent phenotypes. This point is particularly important in orthopedic degeneration, because chondrocytes, osteogenic cells, and disc cells exist in distinct biomechanical and metabolic microenvironments and may therefore rely on different upstream drivers of SASP. Accordingly, mechanism-based intervention should aim to match the dominant regulatory vulnerability of each tissue, which also provides a rationale for combination strategies integrating senolysis, senomorphic suppression, and restoration of intracellular homeostasis (Zhao et al., 2020; Li et al., 2025a; Song Y. et al., 2024; Salminen et al., 2012; Huo et al., 2024; Zhao et al., 2023; Wu Z. et al., 2025; Klepacki et al., 2025) (Figure 2).

### 5.2 Therapeutic strategies targeting SASP

#### 5.2.1 Senolytics (drugs for clearing senescent cells)

Senolytics—drugs that selectively eliminate senescent cells with minimal normal cell impact—are a key SASP-targeted therapeutic strategy (Lagoumtzi and Chondrogianni, 2021; Wu Y. et al., 2022). Cherif et al. first showed Mouse Double Minute 2(MDM2) inhibitor RG-7112 and natural o-Vanillin selectively kill senescent disc cells, reduce SASP, improve disc matrix homeostasis,and alleviate human IVDD (with clinical translation potential) (Cherif et al., 2020). B-Cell Lymphoma 2(Bcl-2) inhibitor ABT263 (Navitoclax) is another effective senolytic: Lim et al. delivered it via PLGA nanoparticles to clear disc senescent cells, reduce inflammatory factors/matrix metalloproteinases, inhibit IVDD, and restore disc structure (Lim et al., 2022); Yang et al. found it also clears skeletal senescent cells to alleviate vitamin D insufficiency-induced OP(30). DQ also exhibits senolytic effects: Wang et al. demonstrated it targets bone marrow senescent cells, restores MSCs, promotes bone regeneration, and improves postmenopausal OP(29). Additionally, natural compound Glycyrrhizin has senolytic activity: Yamada et al. confirmed it alleviates inflammatory bone loss, promotes bone marrow protective sirtuins, inhibits SASP, and holds potential for hip periprosthetic osteolysis (Yamada et al., 2021). ABT263 has been investigated in preclinical studies for age-related musculoskeletal disorders, exhibiting a favorable safety profile in eliminating senescent skeletal cells and attenuating osteoporotic bone loss (Yang et al., 2024). RG-7112 is in preclinical development for IVDD, with *ex vivo* human disc tissue studies validating its selective senescent cell clearance efficacy without off-target matrix damage. These senolytic interventions primarily target the SASP-driven pathogenic mechanisms of senescence spreading, chronic inflammation, and aberrant neurovascular ingrowth (Cherif et al., 2020; Mannarino et al., 2023).

#### 5.2.2 Senomorphics (drugs for inhibiting SASP)

Senomorphics inhibit SASP production/release without directly killing senescent cells (Li et al., 2025a; Mavrogonatou and Kletsas, 2024). JAK inhibitors (typical senomorphics) suppress JAK/STAT to lower SASP; Wu et al. showed they reduce estrogen deficiency-induced SASP in BMSCs, enhance osteogenic differentiation, and improve postmenopausal OP(106). NF-κB inhibitors are another key class: Peng et al. found ubiquitin-editing enzyme A20 restricts NF-κB to protect NP cells from TNF-α-induced senescence (Peng et al., 2020); Chen et al. demonstrated Dendrobine reduces SASP via ROS/NF-κB inhibition, improving OA(64). mTOR inhibitors (e.g., rapamycin) also work: Song et al. found NLRX1-SLC39A7 regulates mitochondrial zinc transport to coordinate mitochondrial dynamics/autophagy, inhibit mTOR, and alleviate NP senescence/SASP(22). Additionally, natural polyphenol resveratrol (anti-inflammatory/antioxidant/anti-aging) improves chondrocyte ER stress, inhibits SASP, and protects joint function (Hecht et al., 2021). JAK inhibitors (e.g., tofacitinib) have completed trials for refractory OA, showing marked suppression of synovial SASP secretion and improved joint function (Paulissen et al. 2025; Koizumi et al., 2015). Rapamycin analogs are under evaluation for age-related OP, demonstrating the ability to restore BMSC osteogenic differentiation by inhibiting mTOR-mediated SASP production (Hou et al., 2025). Senomorphic strategies mainly interrupt SASP-mediated chronic inflammation, immune dysregulation, and ECM catabolism.

#### 5.2.3 Regulatory strategies based on autophagy and gene editing

Autophagy activation is an emerging strategy for regulating SASP. As the primary mechanism for intracellular waste clearance and renewal, autophagy dysfunction is closely associated with cellular senescence and SASP accumulation (Liu et al., 2023). Wu et al. demonstrated that UCHL1 activates CMA by deubiquitinating HSPA8, clears harmful substances, and alleviates NP cell senescence and SASP production (Wu Z. et al., 2025); Cheng et al. confirmed that CMA blockade leads to Phospholipase C Gamma 1(PLCG1) accumulation, causes calcium overload, and promotes NP cell senescence, while PLCG1 knockdown can improve NP cell function (Cheng Z. et al., 2025). Gene- and RNA-directed strategies remain promising but should currently be regarded as exploratory rather than established therapeutic platforms for orthopedic degeneration. Their potential value lies in mechanistic specificity: for example, HIF1A@NNP was designed to regulate disc inflammation and SASP output through autophagy-related pathways in a cell-targeted manner, whereas restoration of miR-325–3p counteracts mechanically induced chondrocyte senescence by modulating the p53/p21 axis (Li et al., 2025a; Zhao et al., 2023). Autophagy activation and gene-/RNA-targeted approaches primarily reverse SASP-induced ECM homeostasis disruption, stem/progenitor cell dysfunction, and senescence spreading (Table 3) (Table 4).

**TABLE 3:** SASP-targeted therapeutic strategies for orthopedic degenerative diseases (preclinical/clinical evidence).

Therapeutic class	Representative agents/Strategies	Targeted molecular pathways/Mechanisms	Efficacy in orthopedic degenerative diseases	Clinical translation stage	Corresponding references
Senolytics (senescent Cell clearance)	RG-7112 plus o-Vanillin	Selective elimination of senescent disc cells and reduction of SASP burden through senolytic activity, including modulation of p53-linked survival signaling	Improves disc matrix homeostasis and reduces the senescence/inflammatory burden in degenerating IVD tissue	Preclinical/*ex vivo* human disc evidence	40, 114
	ABT263 (Navitoclax), including local PLGA-based delivery	Bcl-2-family inhibition with senescent-cell clearance; local delivery may enhance disc specificity	Attenuates vitamin D insufficiency-related osteoporosis, clears senescent skeletal cells, and restores disc structure in IVDD models	Preclinical (animal models); delivery optimization remains preclinical	30, 38
	Dasatinib plus quercetin (DQ)	Senolytic clearance of senescent bone marrow/skeletal cells	Rejuvenates BMSCs, promotes bone regeneration in postmenopausal osteoporosis, and improves subchondral bone-related dysfunction in OA-associated settings	Preclinical in orthopedic disease models; broader senolytic clinical translation remains indirect	29
Senomorphics (SASP suppression)	JAK inhibitors	Suppression of JAK/STAT-driven SASP signaling	Reduces estrogen deficiency-associated SASP in BMSCs and improves osteogenic differentiation in OP, with emerging translational relevance in OA	Orthopedic evidence spans preclinical to early clinical/repurposing discussion	106, 117, 118
	Dendrobine; A20/NF-kappaB modulation	Inhibition of ROS/NF-kappaB or NF-kappaB-centered inflammatory signaling	Reduces SASP output and matrix catabolism in OA and protects NP cells under inflammatory stress	Preclinical	64, 68
	mTOR-modulating approaches (e.g., rapamycin-related pathway control; NLRX1-SLC39A7 axis)	Attenuation of mTOR-linked senescence/SASP programs and restoration of mitochondrial quality control	Alleviates NP-cell senescence/SASP in IVDD and supports preservation of cell homeostasis	Preclinical	22
Autophagy-/Proteostasis-based strategies	NBR1-mediated SRBD1 clearance	Selective autophagic removal of SRBD1 to restrain NP-cell senescence and SASP production	Preserves matrix homeostasis and delays degenerative disc changes	Preclinical	39
​	UCHL1-HSPA8-mediated chaperone-mediated autophagy (CMA)	Enhancement of CMA and intracellular proteostasis, with antagonism of autophagy-dependent ferroptotic stress	Reduces NP-cell senescence/SASP and improves the degenerative microenvironment in IVDD	Preclinical	70, 121
Gene-/RNA-directed or targeted delivery strategies	HIF1A@NNP	Cell-specific nanoparticle-based regulation of disc inflammation and SASP via autophagy-related pathways	Reduces SASP-linked disc inflammation in a tissue-targeted manner	Exploratory preclinical	20
	miR-325–3p restoration	Counteracts mechanically induced chondrocyte senescence via the p53/p21 axis	Suppresses senescence-associated degeneration in mechanically stressed chondrocytes	Exploratory preclinical	65

**TABLE 4:** Mechanism-linked therapeutic strategies targeting SASP in orthopedic degenerative diseases.

Pathogenic mechanism	Main therapeutic class	Representative strategy	Intended effect
Chronic inflammation/immune dysregulation	Senomorphics	JAK inhibitors; dendrobine; A20/NF-kappaB modulation; Imrecoxib-mediated control of macrophage polarization	Suppress inflammatory SASP signaling, reduce macrophage M1-skewing, and interrupt the inflammaging-like feed-forward loop
ECM catabolism	Senomorphics/Autophagy-based strategies	Dendrobine; STAG1/TP53/P21-targeting intervention; NBR1-mediated SRBD1 clearance; UCHL1-HSPA8-CMA activation	Reduce MMP/ADAMTS output, preserve COL2A1/ACAN-related matrix homeostasis, and slow structural tissue breakdown
Senescence spreading	Senolytics/Gene- or RNA-directed approaches	RG-7112 plus o-Vanillin; HIF1A@NNP; miR-325–3p restoration	Decrease the source of pathogenic SASP, block paracrine propagation of senescence, and limit expansion of the degenerative cellular network
Stem/progenitor-cell dysfunction	Senolytics/Senomorphics	Dasatinib plus quercetin; JAK2/STAT3-targeted suppression of BMSC senescence-associated SASP	Restore stem/progenitor-cell fitness, improve osteogenic or reparative potential, and re-establish tissue-maintenance capacity
Aberrant neurovascular remodeling	Senolytics/Targeted local strategies	Senolytic reduction of SASP burden in degenerating disc tissue; local disc-targeted delivery platforms such as PLGA-based senolytic systems or HIF1A@NNP	Reduce pro-angiogenic/pro-inflammatory signaling, limit neurovascular invasion, and weaken pain-associated remodeling in degenerative tissues

### 5.3 Combined intervention strategies and future perspectives

While preclinical evidence supporting SASP-targeted therapy is expanding rapidly, clinical translation remains challenging and is considered a key unresolved issue in this field. First, the SASP is highly heterogeneous across different tissues, disease stages, and senescent cell subtypes, making it difficult to select biomarkers and stratify patients (Kumar et al., 2021; Yang et al., 2026). Second, systemic senolysis may cause off-target toxicity or interfere with beneficial transient senescence, whereas local delivery strategies—though promising for joints and intervertebral discs—still need to overcome obstacles related to retention, penetration, dosing, and long-term safety (Zhang et al., 2026; Peilin et al., 2019). Third, clinically meaningful endpoints should go beyond the molecular suppression of the SASP to include pain relief, structural preservation, physical function, and ideally, biomarker-supported evidence of mechanism engagement (Wang and Guan, 2025; Nusrat et al., 2025). Finally, future clinical trials will likely require a precision framework that integrates tissue-specific biomarkers, imaging, disease stage, and combination regimens combining SASP control with regenerative or biomechanical interventions (Li et al., 2024; Wu H. et al., 2025).

## 6 Conclusion

SASP is best understood not as the initiator of cellular senescence, but as a downstream and central pathogenic effector through which senescent cells drive chronic inflammation, extracellular matrix breakdown, senescence propagation, stem/progenitor-cell dysfunction, and, in selected settings, aberrant neurovascular remodeling in orthopedic tissues (Liu Y. et al., 2024; Zhu et al., 2022). Its biological consequences are shared across diseases but expressed in a tissue- and compartment-specific manner: in OA, SASP coordinates cartilage catabolism, synovitis, and subchondral remodeling; in IVDD, it sustains matrix loss, inflammatory amplification, and neurovascular invasion; and in OP, it contributes to bone-remodeling imbalance and skeletal stem-cell dysfunction (Yang et al., 2024; Zhou et al., 2025; Mannarino et al., 2025; Zhao et al., 2025; Wu et al., 2020). Therapeutic development should therefore move toward mechanism-linked and clinically stratified intervention. Senolytics reduce the cellular source of SASP, senomorphics attenuate inflammatory and catabolic signaling, autophagy-based strategies improve intracellular homeostasis, and gene-/RNA-targeted approaches may eventually enable precision regulation in selected tissues. However, true clinical translation will depend on solving problems of target specificity, delivery, biomarker-guided patient selection, and safety, while integrating SASP modulation with structural repair and functional outcome assessment. A more precise understanding of tissue-specific SASP programs may thus provide a realistic path toward translationally meaningful therapies for age-related orthopedic degeneration.

Future research should focus on: elucidating SASP’s molecular regulatory networks in different orthopedic tissues (clarifying tissue/disease specificity), developing more precise/effective SASP-targeted drugs (enhancing specificity/safety), exploring multi-target combined interventions (synergistically regulating SASP), integrating tissue engineering/stem cells for SASP regulation-tissue regeneration regimens, and conducting preclinical/clinical studies to verify efficacy/safety (Peng et al., 2020). With deeper understanding of SASP mechanisms and new technologies, its targeted precision therapy may breakthrough orthopedic degenerative disease treatment, improve patient quality of life, reduce medical burden, and become a new strategy via interdisciplinary integration and basic-clinical translation (Ling et al., 2025).

## Glossary

SASP: Senescence-Associated Secretory Phenotype
OA: Osteoarthritis
IVDD: Intervertebral Disc Degeneration
OP: Osteoporosis
ECM: Extracellular Matrix
BMSCs: Bone Marrow Mesenchymal Stem Cells
NP: Nucleus Pulposus
AF: Annulus Fibrosus
EP: Cartilaginous Endplate
NF-κB: Nuclear Factor Kappa-Light-Chain-Enhancer of Activated B Cells
MAPK: Mitogen-Activated Protein Kinase
MMPs: Matrix Metalloproteinases
ADAMTS: A Disintegrin and Metalloproteinase with Thrombospondin Motifs
IL: Interleukin
TNF-α: Tumor Necrosis Factor-Alpha
ROS: Reactive Oxygen Species
DDR: DNA Damage Response
mTOR: Mechanistic Target of Rapamycin
JAK/STAT: Janus Kinase/Signal Transducer and Activator of Transcription
CMA: Chaperone-Mediated Autophagy
sEVs: Small Extracellular Vesicles
EMT: Epithelial-Mesenchymal Transition
LIPUS: Low-Intensity Pulsed Ultrasound
HIF1A: Hypoxia-Inducible Factor 1-Alpha
AMPK/GSK3β: Adenosine Monophosphate-Activated Protein Kinase/Glycogen Synthase Kinase 3β
ATM: Ataxia-Telangiectasia Mutated
ATF3: Activating Transcription Factor 3
AP-1: Activator Protein-1
Bcl-2: B-Cell Lymphoma 2
CCL2: C-C Motif Chemokine Ligand 2
CCL5: C-C Motif Chemokine Ligand 5
CB2R: Cannabinoid Receptor Type 2
Cd: Cadmium
CeNP: Cerium Oxide Nanoparticles
CRLF1/CLCF1: Cytokine Receptor-Like Factor 1/Cardiotrophin-Like Cytokine Factor 1
CX43: Connexin 43
ERK1/2: Extracellular Signal-Regulated Kinase 1/2
FGF: Fibroblast Growth Factor
FOXO3: Forkhead Box O3
H3K9me3: Histone H3 Lysine 9 Trimethylation
HSPA8: Heat Shock Protein Family A (Hsp70) Member 8
HPDLCs: Human Periodontal Ligament Cells
IKKβ: Inhibitor of Nuclear Factor Kappa-B Kinase Subunit Beta
JNK: c-Jun N-Terminal Kinase
LOX-1: Lectin-Like Oxidized Low-Density Lipoprotein Receptor 1
MAC: Membrane Attack Complex
MDM2: Mouse Double Minute 2
miR-325-3p: MicroRNA-325–3p
NBR1: Next to BRCA1 Gene 1
NLRX1: NOD-Like Receptor X1
Nrf2: Nuclear Factor Erythroid 2-Related Factor 2
ox-LDL: Oxidized Low-Density Lipoprotein
p16Ink4a: Cyclin-Dependent Kinase Inhibitor 2A
p21: Cyclin-Dependent Kinase Inhibitor 1A
p53/TP53: Tumor Protein 53
PCB2: Procyanidin B2
PI3K/AKT: Phosphoinositide 3-Kinase/Protein Kinase B
PLGA: Poly(Lactic-Co-Glycolic Acid)
PLCG1: Phospholipase C Gamma 1
PTH(1–34): Parathyroid Hormone (1–34)
P2X7: P2X Purinoceptor 7
RB: Retinoblastoma Protein
RANKL: Receptor Activator of Nuclear Factor Kappa-B Ligand
SIRT6: Sirtuin 6
Smad3: Mothers Against Decapentaplegic Homolog 3
SRBD1: Serine-Rich RNA Binding Domain 1
STAG1: Stromal Antigen 1
SUV39H1: Suppressor of Variegation 3-9 Homolog 1
TGF-β: Transforming Growth Factor Beta
UCHL1: Ubiquitin C-Terminal Hydrolase L1
VSMC: Vascular Smooth Muscle Cell
YAP: Yes-Associated Protein
Zn: Zinc
γH2AX: Phosphorylated Histone H2AX
Ki67: Ki67 Proliferation Marker
COX-2/PGE2: Cyclooxygenase-2/Prostaglandin E2
COL2A1: Collagen Type II Alpha 1 Chain
ACAN: Aggrecan
TLR2: Toll-Like Receptor 2
TLR4: Toll-Like Receptor 4
MyD88: Myeloid Differentiation Primary Response 88
FAK: Focal Adhesion Kinase
NFATc1: Nuclear Factor of Activated T-Cells Cytoplasmic 1
DQ: Dasatinib + Quercetin
ABT263: Navitoclax
RG-7112: MDM2 Inhibitor RG-7112
STAT3: Signal Transducer and Activator of Transcription 3
